# Senescent and disease-associated microglia are modifiable features of aged brain white matter

**DOI:** 10.21203/rs.3.rs-3467812/v1

**Published:** 2023-10-30

**Authors:** Chase M. Carver, Paul T. Gomez, Sonia L. Rodriguez, Jennifer M. Kachergus, Yi Liu, Ji Shi, Tommy Tran, Liguo Wang, Simon Melov, E. Aubrey Thompson, Marissa J. Schafer

**Affiliations:** 1 Department of Physiology and Biomedical Engineering, Mayo Clinic, Rochester, MN, USA; 2 Robert and Arlene Kogod Center on Aging, Mayo Clinic, Rochester, MN, USA; 3 Department of Cancer Biology, Mayo Clinic, Jacksonville, FL, USA; 4 Department of Quantitative Health Sciences, Mayo Clinic, Jacksonville, FL, USA; 5 Buck Institute for Research on Aging, Novato, CA, USA; 6 Division of Computational Biology, Mayo Clinic College of Medicine, Rochester, MN, USA; 7 Department of Neurology, Mayo Clinic, Rochester, MN, USA

**Keywords:** aging, microglia, white matter, senotherapeutics, neuroinflammation

## Abstract

Brain white matter tracts undergo structural and functional changes linked to late-life cognitive decline, but the cellular and molecular contributions to their selective vulnerability are not well defined. In naturally aged mice, we demonstrate that senescent and disease-associated microglia (DAM) phenotypes converge in hippocampus-adjacent white matter. Through gold-standard gene expression and immunolabeling combined with high-dimensional spatial mapping, we identified microglial cell fates in aged white matter characterized by aberrant morphology, microenvironment reorganization, and expression of senescence and DAM markers, including galectin 3 (GAL3/*Lgals3*), B-cell lymphoma 2 (*Bcl2*), and cyclin dependent kinase inhibitors, including *Cdkn2a/p16*^*ink4a*^. Pharmacogenetic or pharmacological targeting of *p16*^*ink4a*^ or BCL2 reduced white matter GAL3+ DAM abundance and rejuvenated microglial fimbria organization. Our results demonstrate dynamic changes in microglial identity in aged white matter that can be reverted by senotherapeutic intervention to promote homeostatic maintenance in the aged brain.

## INTRODUCTION

Aging increases risk for cognitive decline and neurodegenerative conditions, including Alzheimer’s disease, yet the cascades of cellular and molecular events that confer brain dysfunction in aging remain unclear. The hippocampal formation is a central mediator of learning and memory and is age-vulnerable. It is comprised of a cortical gray matter infolding directly abutted by dense white matter tracts, the corpus callosum and fimbria-fornix. The Papez circuit is a memory- and emotion-related pathway through which cortical signals are transmitted through the hippocampus and fimbria-fornix to the hypothalamus, thalamus, and cingulate gyrus^1^. Imaging studies reveal that white matter dysfunction, and particularly changes in fimbria-fornix, may precede cortical gray matter dysfunction, representing an early cortico-limbic disruption underlying memory dysfunction in the progression of cognitive decline^2,3,4,5^. Cell-specific molecular mediators of aging are potential intervention opportunities for preserving brain health and preventing neuropathogenesis in aging.

Inflammatory and senescent fates have emerged as maladaptive cellular trajectories across aging tissues. In the aged and neurodegenerative brain, DAM accumulate and exhibit downregulation of homeostatic genes (*Csf1r, Cx3cr1, Hexb, P2ry12, Tmem119*) and upregulation of genes encoding factors involved in inflammation, immunoregulation, neurodegeneration, and lysosomal stress (*Apoe, B2m, Ccl2, Fth1, H2-Ab1, Itgax, Lgals3)*^6,7,8,9^. The APOE-TREM2 pathway represents a critical regulator of the switch from homeostatic to DAM-like microglia^10,11^. Functional consequences of DAM include inflammatory disruption of brain microenvironments^12,13,14^. Microglia in white matter fulfill overlapping and distinct functional roles relative to grey matter, as defined by biochemical interactions with densely myelinated fiber tracts and neurons, respectively. White-matter associated microglia (WAM) share DAM features as well as varying expression patterns, characterized by cell-type proximity and functional pathways such as iron regulation and lipid metabolism^15,16,17,18^. The delineation of DAM and WAM phenotypes in distinct aged brain structures and their functional consequences are crucial next steps for determining the mechanistic influence of microglia states in the aged brain.

DAM are inherently defined by an altered homeostatic state, and we recently discovered that DAM also represent a molecular senescent state. The gene expression profile of inflammatory senescent DAM cells is characterized by expression of cyclin-dependent kinase inhibitors (*Cdkn2a/p16*^*ink4a*^, *Cdkn1a/p21*^*Cip1*^), chemoattractant factors (*Ccl2-5, Spp1*), pro-survival factors (*Bcl2*), canonical DAM factors (*Apoe, Itgax*), and lysosomal stress factors (*Lgals3*)^19,20^. GAL3, encoded by *Lgals3,* binds to β-galactosidase on stressed lysosomes^21,22,23^, and the presence of senescence-associated β-galactosidase has long been a proxy for senescent cell detection^24^. GAL3/*Lgals3* is also a well-established DAM and WAM factor that interacts with microglial receptors involved in neuroinflammation, contributes to microglia-activated remodeling of myelin, and is linked to cognitive dysfunction^25,26,27,28,29^. We propose GAL3/*Lgals3* is a critical biomarker of senescent DAM in the aged brain.

The last decade’s pivotal advances in characterizing aged brain cell fates, including DAM, WAM, and senescent identities were accomplished, in part, through single-cell RNA sequencing and related approaches that interrogated cell suspensions without spatial context. A new era of biology is upon us, marked by novel technologies that permit high-dimensional mapping of cellular and molecular states with preserved spatial context. Importantly, two recent seminal studies that broadly investigated aged brain cell signatures by spatial transcriptomics identified white matter as distinctly altered in aging^30,31^.

Here, we sought to investigate modifiable senescent and DAM identities in age-vulnerable hippocampal gray and white matter in naturally aged mice. Through use of three emerging spatial molecular imaging platforms, together with gold-standard gene expression and imaging techniques, we dynamically mapped senescence and DAM cell fates in their well-defined microenvironments. We discovered DAM populations harboring senescent phenotypes accumulate in aged white matter. By applying pharmacogenetic and pharmacological strategies to target distinct factors activated in senescent cells, *p16*^*ink4a*^ or BCL2, we demonstrate the ability to reduce age-increased senescence and DAM markers, including *p16*^*ink4a*^, GAL3, and APOE, and restore cellular organization in aged white matter to a more youthful state. These microglial atlases and mechanistic relationships reveal both overlapping and distinct senescent and DAM fates as prominent features of aged white matter that can be partially rejuvenated by senotherapeutic intervention, which may exert beneficial influence on cognition in aging.

## RESULTS

### Expression of DAM and senescence genes, including *p16*^*ink4a*^ and *Lgals3,* increase in aged brain white matter

**Figure 1.**
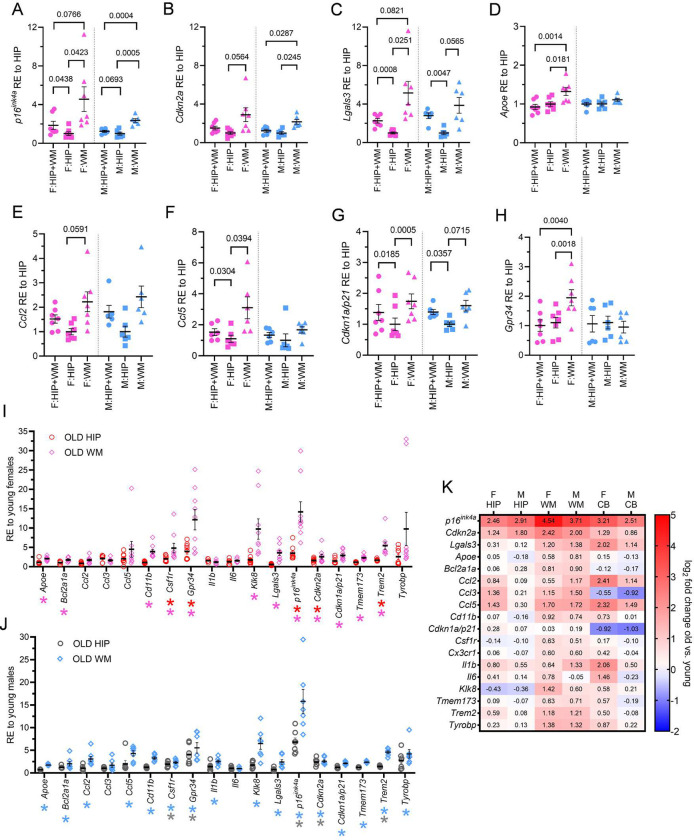
Senescent and disease-associated microglial gene expression increase in aged brain white matter. (A-H) Summarized are RT-PCR relative expression (RE) values from old female (pink) and old male (blue) tissues of hippocampus white matter (HIP+WM, circles), hippocampus only (HIPP, squares), and white matter only (WM, triangles). Values are normalized relative to HIP expression per sex. Comparisons are shown for (A) *p16*^*ink4a*^, (B) *Cdkn2a*, (C) *Lgals3*, (D) *Apoe*, (E) *Ccl2*, (F) *Ccl5*, (G) *Cdkn2a/p21*, and (H) *Gpr34*. p values denote one-way ANOVA with multiple comparisons correction. (I) Shown are female RT-PCR expression from old hippocampus (OLD HIP, red circles) and old white matter (OLD WM, pink diamonds) normalized relative to expression of the same tissue from young females. (J) Shown are the male RT-PCR RE values from old hippocampus (OLD HIP, grey circles) and old white matter (OLD WM, blue diamonds) normalized relative to expression of the same tissue from young males. (I-J: Unpaired t-tests with FDR correction, *q < 0.05 vs. young tissue, n = 6–9 mice). (K) Shown are the log_2_ fold-changes in RT-PCR gene expression between young and old brain regions of HIP, hippocampus-adjacent WM, and cerebellum (CB) in female (F) and male (M) mice. (Statistics are published **Extended Data Table 1**, n = 5–8 mice). Bars represent mean ± S.E.M.

We previously demonstrated that the aged female hippocampal region harbors increased abundance of DAM and senescence markers, including *Cdkn2a/p16*^*ink4a*^ and *Lgals3*^20^. We hypothesized that hippocampal-adjacent white matter accumulates senescent DAM markers in aging. For direct comparison, we microdissected hippocampal gray matter from surrounding fimbria-fornix and corpus callosum white matter from one brain hemisphere and extracted the combined hippocampus and white matter from the other hemisphere of aged female and male brains. We assessed expression of senescence and microglial genes among the microdissections (Figure 1). We distinguish between detection of both *Cdkn2a* (splice variant *p19*^*arf*^ and variant *p16*^*ink4a*^ together, heretofore referred to as *Cdkn2a*) and *p16*^*ink4a*^ detection alone. *p16*^*ink4a*^ (Fig. 1A)*, Cdkn2a* (Fig. 1B), and *Lgals3* (Fig. 1C) expression were higher in white matter as compared to the hippocampus. In female mice, expression levels of *Apoe* (Fig. 1D), *Ccl2* (Fig. 1E), *Ccl5* (Fig. 1F), *Cdkn1a/p21* (Fig. 1G), and *Gpr34* (Fig. 1H) were significantly or trending higher in white matter. Enrichment of senescent and DAM biomarkers in aged white matter led us to investigate young versus aged expression patterns (Fig. 1I–J). Relative to young counterparts, *p16*^*ink4a*^*, Cdk2na, Csf1r, Gpr34, and Trem2* expression increased in white matter and hippocampus from old male and female mice. *Apoe, Bcl2a1a, Cd11b, Klk8, Lgals3, Cdkn1a/p21,* and *Tmem173* expression increased in old female and male white matter. *Ccl2, Ccl5, Il1b,* and *Tyrobp* additionally increased in aged male white matter (Fig. 1J). In aged female and male mice, senescence and DAM gene expression induction was more pronounced in hippocampus-adjacent white matter, relative to hippocampus. To provide regional context to white matter-derived changes in aging, we compared young verus old gene expression in the hippocampus, white matter, and cerebellum. Age-dependent increases in *p16*^*ink4a*^*, Cdkn2a, Lgals3, Apoe, Bcl2a1a, Cd11b, Csf1r*, *Klk8, Tmem173, Trem2,* and *Tyrobp* were pronounced in old white matter (Figure 1K). This suggests that white matter is particularly susceptible to late-life changes in senescence and DAM transcriptional signatures.

### GAL3-positive myeloid cells accumulate in the hippocampal-adjacent white matter of old mice

**Extended Data Figure 1.**
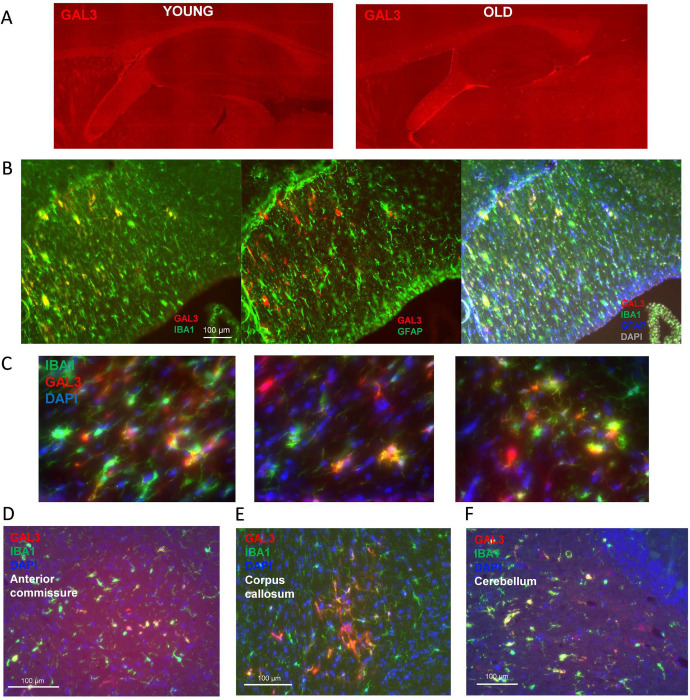
GAL3 positive microglia accumulate in aged white matter. (A) Immunoreactivity for GAL3 in hippocampus and adjacent white matter in young (left) and old (right) female mouse brains. (B, left) GAL3 (red) and IBA1 (green) immunoreactivity in an old mouse fimbria. (B, center) GAL3 (red) and GFAP (green) immunoreactivity in an old mouse fimbria. (B, right) GAL3 (red), IBA1 (green), and GFAP (blue) immunoreactivity in an old mouse fimbria, with DAPI nuclear stain in grey. (C) Representative magnified images of GAL3+ and IBA1+ cells from an old mouse fimbria, with DAPI nuclear stain in blue. (D-F) GAL3 and IBA1 immunoreactivity in (D) corpus callosum, (E) anterior commissure and (F) deep cerebellar nuclei fiber tracts of an old mouse.

Based on the observation that senescent DAM cells express *Lgals3*/GAL3^20, 32^, we investigated the abundance of GAL3+ cells across the hippocampus and adjacent white matter in young and old brain slices (Extended Data Fig. 1). In young mice, GAL3+ cells were rare. We observed diverse immunoreactivity of GAL3+ cells over multiple regions of the old brain (Extended Data Fig. 1A) with dense localization in white matter tracts, especially the fimbria (Extended Data Fig. 1A). In aged fimbria, GAL3+ colocalized with myeloid/microglial marker IBA1 but not with astrocyte marker GFAP (Extended Data Fig. 1B–C). Like the fimbria, the corpus callosum and anterior commissure exhibited strong GAL3+ immunoreactivity (Extended Data Fig. 1D–E). IBA1 and GAL3 also colocalized in deep cerebellar nuclei (Extended Data Fig. 1F). Through immunofluorescent imaging and gene expression, we demonstrate increased abundance of senescent and DAM markers in aged white matter, an effect that was pronounced in the fimbria of old females. We next used three emerging spatial profiling methods to deeply investigate the molecular identities of aged microglia in distinct limbic microenvironments.

### Imaging mass cytometry (IMC) demonstrates microglia positive for senescent and DAM markers are abundant in the aged fimbria

**Figure 2.**
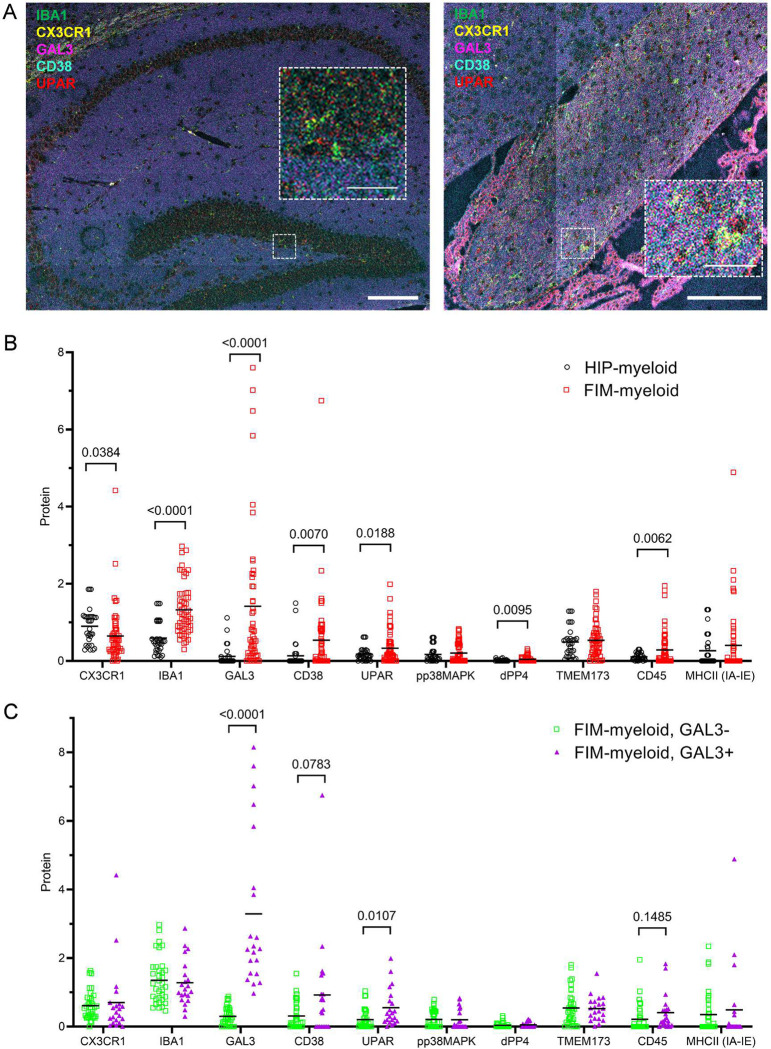
IMC reveals protein signatures of senescent and DAM are increased in aged fimbria. (A) Representative reconstructed IMC in old female hippocampus (left) and fimbria with protein labels for IBA1 (green), CX3CR1 (yellow), GAL3 (magenta), CD38 (cyan), and UPAR (red), scale bars = 200 μm. (B) Summarized are background-subtracted protein values for IBA1/CX3CR1+ cells from the hippocampus and fimbria of old brains. (C) Summarized are background-subtracted protein values for fimbria-specific IBA1/CX3CR1+ cells stratified into GAL3− versus GAL3+. P-values denote unpaired t-tests with multiple comparisons correction. Bars represent mean ± S.E.M, n = 17–31 cells from 4 mice.

We implemented IMC, which combines immunolabeling and time-of-flight mass spectrometry, to map and quantify ten protein markers with one μm spatial resolution in the aged brain (Fig. 2). We measured colocalization of senescent and DAM markers (GAL3, TMEM173, MHCII, UPAR, dPP4, phospho-p38MAPK, CD38) with IBA1 and CX3CR1 as myeloid markers and CD45 as immune markers (Fig. 2A). Fimbria myeloid cells had greater intensity of IBA1, GAL3, CD38, UPAR, dPP4 (CD26), and CD45, relative to hippocampal myeloid cells, which exhibited greater CX3CR1 protein levels (Fig. 2B). Stratification of GAL3+ and GAL3− cells in fimbria showed that GAL3+ cells harbored trending or significantly higher levels of CD38 and UPAR than GAL3−, signifying the delineation of a population of senescent microglia based on GAL3 immunoreactivity (Fig. 2C). These protein-based results reinforce the observation that aged fimbria accumulates a high burden of senescent and DAM markers, including GAL3 and UPAR.

### GeoMx digital spatial profiling reveals enrichment of DAM and senescent microglia in aged white matter

**Figure 3.**
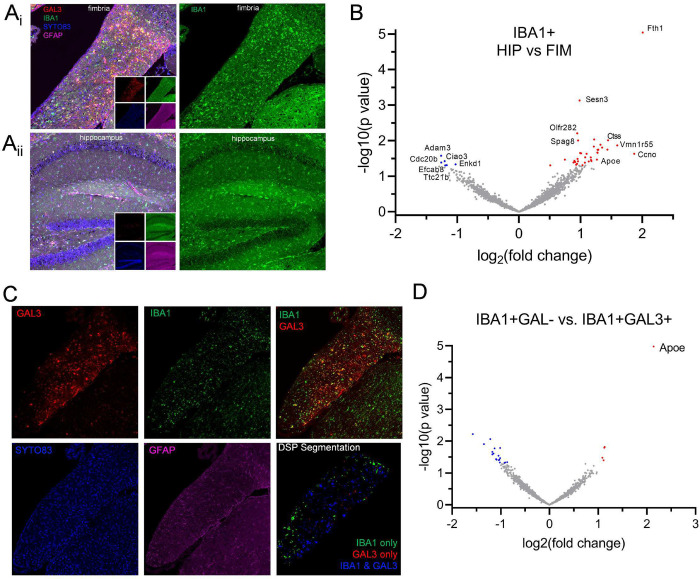
GeoMx digital spatial profiling demonstrates conserved DAM and novel genes increase in the aged fimbria. (A) Representative immunostaining of sections from (A_i_) fimbria and (A_ii_) hippocampus with labels for GAL3 (red), IBA1 (green), SYTO83 (blue), and GFAP (pink) in old mouse brain sections used for spatial profiling. Right panels show IBA1 staining alone in green. B) Volcano plot for genes in comparison of IBA1+ AOIs in hippocampus versus fimbria. Blue points indicate higher enrichment in hippocampus, red points indicate enrichment in fimbria (DESeq2, p < 0.05, n = 8–15 sections sampled from 5 mice). (C) Immunostaining and segmentation of fimbria for distinguishing IBA1+GAL3− and IBA1+GAL3+ cells. (D) Volcano plot for gene comparison of IBA1+GAL3− versus IBA1+GAL3+ cellular AOIs in old fimbria demonstrating *Apoe* is distinctly expressed in IBA+GAL3+ microglia (DESeq2, p < 0.05, n = 15 sections sampled from 5 mice).

We next examined microglial transcriptional signatures specific to hippocampus and fimbria of old female mice using GeoMx digital spatial profiling. We segmented hippocampus and fimbria regions with positive selection based on immunostaining with GAL3, IBA1, and SYTO83 and negative selection by GFAP (Fig. 3A). First, we compared IBA1+ cell populations in the hippocampus versus fimbria (Fig. 3B). DAM genes *Fth1, Ctss, and Apoe* and putative senescence-related genes, *Ccno* and *Sesn3*, were enriched in IBA1+ cells of fimbria. *Adam3, Ciao3, Cdc20b, Enkd1, Ttc21b, and Efcab8* transcripts were enriched in hippocampal IBA1+ cells (**Extended Data Table 2**). We next compared transcriptional profiles of IBA1+GAL3+ and IBA1+GAL3− microglia specifically residing in aged fimbria (Fig. 3C–D). *Apoe* was the most abundantly expressed gene in IBA1+GAL3+ cells, relative to IBA1+GAL3− cells. Thus, digital spatial profiling revealed new molecular targets enriched in aged fimbria that are linked to increased DAM gene expression and confirmed *Apoe* expression in aged fimbria GAL3+ microglia.

### CosMx spatial molecular imaging defines a mosaic of aged microglial identities, including white matter localized senescent and DAM cells

**Figure 4.**
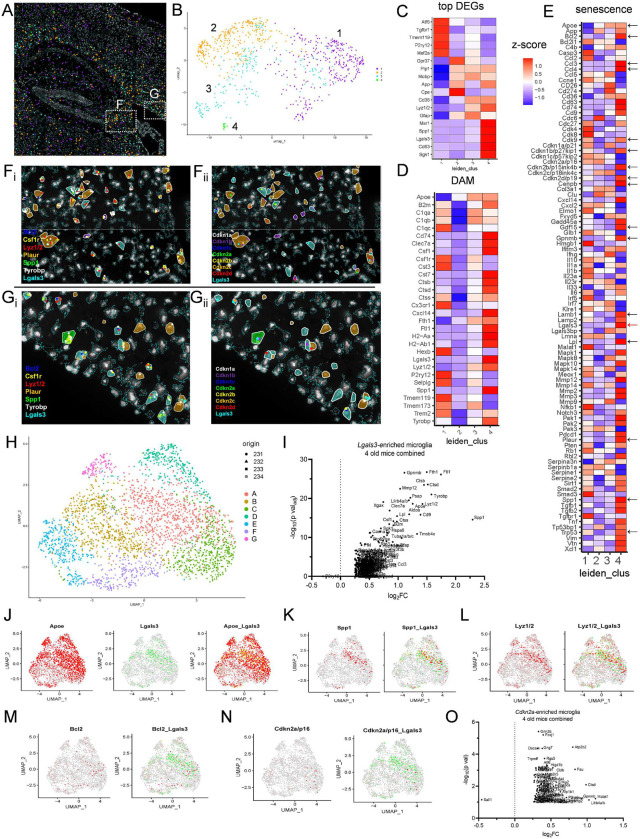
CosMx spatial molecular imaging resolves diverse cell-specific DAM and senescent microglial identities in aged brain. (A) Representative CosMx image of microglial centroid spatial localizations across hippocampus, cortex, thalamus, and white matter regions in a 24-month-old female mouse brain. (B) UMAP Leiden clustering of 790 microglia as represented spatially in A, separated into four clusters generally delineated as cluster 1: grey matter-resident microglia, and clusters 2–4: white matter-resident microglia. Clusters were created from 1000-plex gene features with no spatial information input as a factor. (C) Heatmap of the five top differentially expressed genes from clusters 1–4 as shown in B. (D) Heatmap panel of normalized DAM genes from clusters 1–4 as shown in B. (E) Heatmap panel of normalized senescence genes from clusters 1–4 as shown in B. Arrows indicate key senescence markers detected in cluster 4 microglia. (C-E) Color-values per heatmap cell represent normalized z-scores from Gini coefficient analysis, blue denotes lower and red denotes higher relative expression. (F_i_, left) Overlay of polygonal cell segmentation of white matter fimbria microglia with CosMx transcript localizations *Bcl2* (blue), *Csf1r* (yellow), *Lgals3* (cyan), *Lyz1/2* (red), *Plaur* (orange), *Spp1* (green), and *Tyrobp* (white). (F_ii_, right) Representative transcript localizations for cyclin-dependent kinase inhibitors *Cdkn1a/p21* (white), *Cdkn1b/p27kip1* (purple), *Cdkn1c/p57kip2* (blue), *Cdkn2a/p16* (green), *Cdkn2b/p15ink4b* (yellow), *Cdkn2c/p18ink4c* (orange), *Cdkn2d/p19* (red), and *Lgals3* (cyan) in same section area as in the left panel. (G_i_, left) Overlay of polygonal cell segmentation of white matter corpus callosum microglial localizations as in F_i_. (G_ii_, right) Representative transcript localizations for cylcin-dependent kinase inhibitors as in F_ii_. (H) UMAP of pooled microglia from four old mouse brains, grouped by shape (denoting mouse ID of origin #231-234) and grouped by color (denoting Leiden cluster assignment). (I) Volcano plot of genes upregulated in *Lgals3-*enriched microglia (with 2 or greater *Lgals3* transcript counts per cell), compared to microglia with 1 or no counts of *Lgals3* per cell. (J) Feature plot mapped across pooled microglia as in H, for *Apoe* (red) and *Lgals3* (green) and *Lgals3 + Apoe* (yellow). (K) Feature plot mapped across pooled microglia as in H for *Spp1* (red) and *Lgals3 + Spp1* (yellow). (L) Feature plot mapped across pooled microglia as in H for *Lyz1/2* (red) and *Lgals3 + Lyz1/2* (yellow). (M) Feature plot mapped across pooled microglia as in H for *Bcl2* (red) and *Lgals3 + Bcl2* (yellow). (N) Feature plot mapped across pooled microglia as in H for *Cdkn2a* (red) and *Lgals3 + Cdkn2a* (yellow). (O) Volcano plot of genes upregulated in *Cdkn2a*-enriched microglia (with 2 or greater *Cdkn2a* transcript counts per cell), compared to microglia with 1 or fewer count of *Cdkn2a* per cell.

We used CosMx spatial molecular imaging to define microglial transcriptional signatures at single-cell resolution in the hippocampus, corpus collosum, fimbria, cortex, dorsal thalamus, and choroid plexus of the aged female mice (Fig. 4A). We developed a 50-gene custom panel of senescence-associated genes based on a two-step process of (1) selection of genes from published senescence profiles^20, 33, 34, 35^ and (2) confirmation of brain cell expression in aged brain single-cell RNA sequencing and spatial transcriptome datasets^30, 36, 37^, which we integrated in tandem with the 950-plex CosMx mouse neuroscience panel (**Extended Data Table 3**). Microglia were identified based on previously established marker selection including *Csf1r*, *Ctss*, *Cx3cr1*, *Hexb*, *Selplg*, *Itgam*, *P2ry12, Tmem119*, and *Trem2*^38,39^. From a single old female brain section, we identified four microglia clusters of 780 cells defined by UMAP-based Leiden clustering (Fig. 4A–B). Despite a clustering process based entirely on gene expression, clusters localized to distinct spatial regions. Cluster 1 localized to cortex, hippocampus, and thalamus, and clusters 2, 3, and 4 primarily localized to corpus callosum and fimbria (Fig. 4A–B). White matter-defined clusters 2, 3, and 4 contained oligodendrocyte transcripts *Gpr37, Plp1, Mobp, App, and/or Cpe*, defining close proximity, or prior engulfment of, oligodendrocyte-lineage cells. Cluster 3 was enriched for *Gfap*, indicative of proximity, or engulfment of, astrocyte-lineage cells. Clusters 3 and 4 shared higher expression of *Cd36* and *Lyz1/2*, whereas cluster 4 cells were rarer and defined by *Msr1, Spp1*, *Lgals3, Cd63*, and *Sgk1* (Fig. 4C). Cluster 1 was characterized by low expression of *Apoe* and *Fth1* and high expression of genes *C1qc, Csf1r, Cst3, Cx3cr1, Hexb, P2ry12, Selplg, Tmem119* and *Tmem173,* suggestive of a homeostatic population (Fig. 4D). DAM transcripts *B2m*, *Cd74*, *Clec7a, Csf1, Cst7, Ctsb, Ctsd, Cxcl14, Fth1, Ftl1, H2-Aa, H2-Ab1*, and *Tyrobp* were distinctly higher in white matter-localized cluster 4 (Fig. 4D).

**Extended Data Figure 2.**
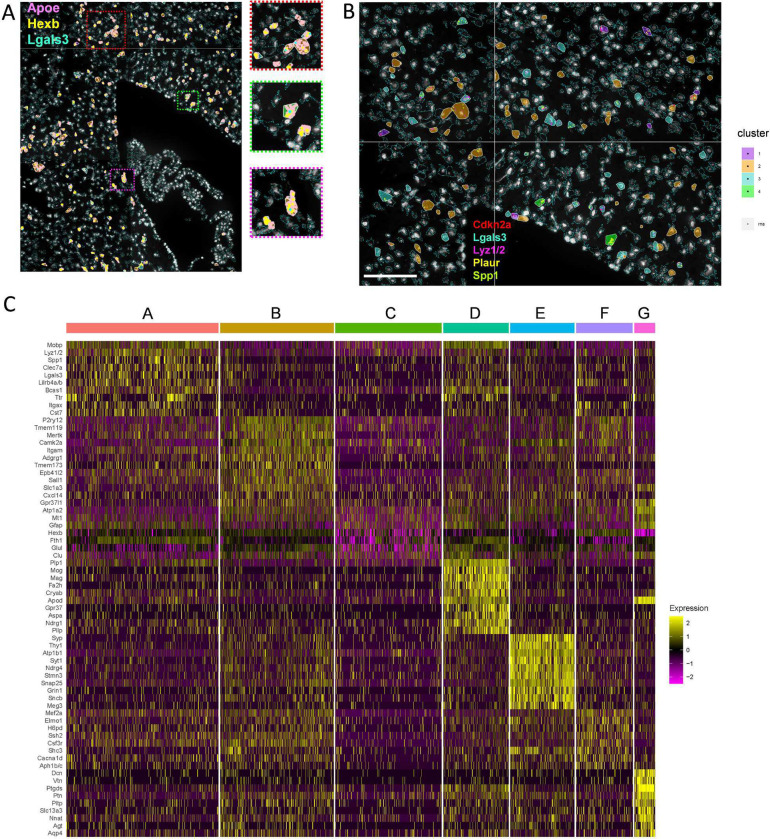
CosMx transcript localization and single-cell cluster differentiation profiles for DAM and senescent microglia. Related to figures 4 and 5: (A) Representative CosMx transcript localizations overlayed onto microglia cells for *Apoe* (red), *Lgals3* (cyan), *Hexb* (green) from the section shown in Figure 4A. Outset panels show specific *Apoe*-concentrated localizations in white matter. (B) Representative CosMx image of *Cdk2na* (red), *Lgals3* (cyan), *Lyz1/2* (magenta), *Plaur* (yellow), and *Spp1* (green) transcript localizations in microglia within white matter. Scale bar, 100 μm. (C) Single-cell heatmap of top highly-expressed genes across pooled microglia from four old brains shown in Figure 4H, organized by cluster identity A-G.

We detected marked diversity in the senescence panel among microglial populations. However, cluster 4 demonstrated the greatest number of highly-expressed senescence markers including *Apoe*, *Bcl2, Ccl3-4, Cdk9, Cdkn1b, Cdkn2b, Cdkn2d, Gdf15, Gpnmb, Lamb1, Lgals3, Lpl, Mmp2-3, Pdcd1, Plaur, Sirt1, Spp1, Tgfb1, Tgfb2,* and *Trp53* (Fig. 4E). We previously discovered that senescence and DAM-associated markers *Cdkn2a/p16*^*ink4a*^ and *Lgals3* were enriched in the same aged brain myeloid population. Surprisingly, clusters 1 and 2 demonstrated relatively high *Cdkn2a* expression compared to clusters 3 and 4 (Fig 4E). While we found rare cells that expressed both *Lgals3* and *Cdkn2a*, the *Lgals3*-enriched cluster 4 had a lower incidence of *Cdkn2a* transcript yet higher expression of *Cdkn1b, Cdkn2b,* and *Cdkn2d*. Spatially, *Cdnk2a*-containing and/or *Lgals3-*containing microglia were frequently in close proximity to microglia harboring other senescent and DAM markers, including *Lyz1/2, Plaur, Spp1*, *Tyrobp*, and cyclin-dependent kinase inhibitor genes (Fig. 4F–G; Extended Data Figure 2A–B). This demonstrates regional conservation, yet subpopulation heterogeneity of microglial fates harboring senescent profiles and reveals a white matter-localized subpopulation as a dominant DAM and senescence-associated microglial population in the aged brain.

**Extended Data Figure 3.**
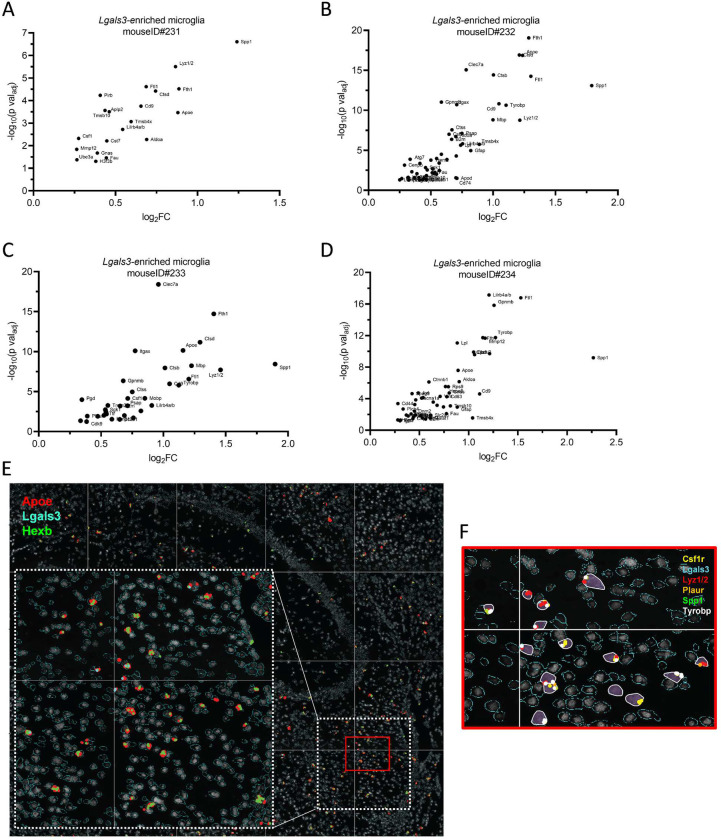
CosMx reveals conservation and heterogeneity of *Lgals3*+ single-cell gene expression profiles across aged brain samples. Related to figure 4 and 5: Gene expression volcano plots derived from *Lgals3-enriched* microglial clusters separated by each mouse brain (A)ID#231, (B)ID#232, (C)ID#233, (D) ID#234. (E) Shown are representative *Apoe* (red), *Lgals3* (cyan), and *Hexb* (green) CosMx transcript localizations in white matter microglia. Inset shows specific *Apoe*-concentrated localizations in fimbria. (F) Magnified outset of DAM and senescence transcripts expressed in fimbria microglia for *Csf1r* (yellow), *Lgals3* (cyan), *Lyz1/2* (red), *Plaur* (orange), *Spp1* (green), and white *(Tyrobp*), from the section depicted in red in E.

We repeated the same CosMx microglial analysis pipeline in three additional aged female brains and merged the data from the four biological replicates to explore signal conservation and diversity in the spatial context. Leiden clustering rendered seven microglial clusters A-G from the pooled four-brain comparison of 2620 total microglial cells (Fig. 4H; Extended Data Figure 2C). Clusters A, C, and D exhibited the highest incidence of DAM expression, similar to the profile of the single-brain cluster 4 above. Across all pooled clusters A-G, we confirmed DAM- and senescence-associated genes *Apoe, Ctsb, Ctsd, Cd9, Cd63, Csf1, Fth1, Ftl1, Gpnmb, Itgax, Lilrb4a/b, Lyz1/2*, *Lpl*, *Mmp12,* and *Spp1* were increased in *Lgals3*-expressing microglia (Fig. 4I), which were also reflected in analysis of *Lgals3*-expressing microglia from each of the four individual brains (Extended Data Figure 3A–D, **Extended Data Table 3**). The microglia clusters A and C harbored the greatest frequency of *Lgals3* cells and demonstrated overlap of greater frequency and/or higher intensity expression of *Apoe* (Fig. 4J), *Spp1* (Fig. 4K)*, Lyz1/2* (Fig. 4L), *Bcl2* (Fig. 4M), and *Cdkn2a* (Fig. 4N, Extended Data Figure 3E–F).

**Extended Data Figure 4.**
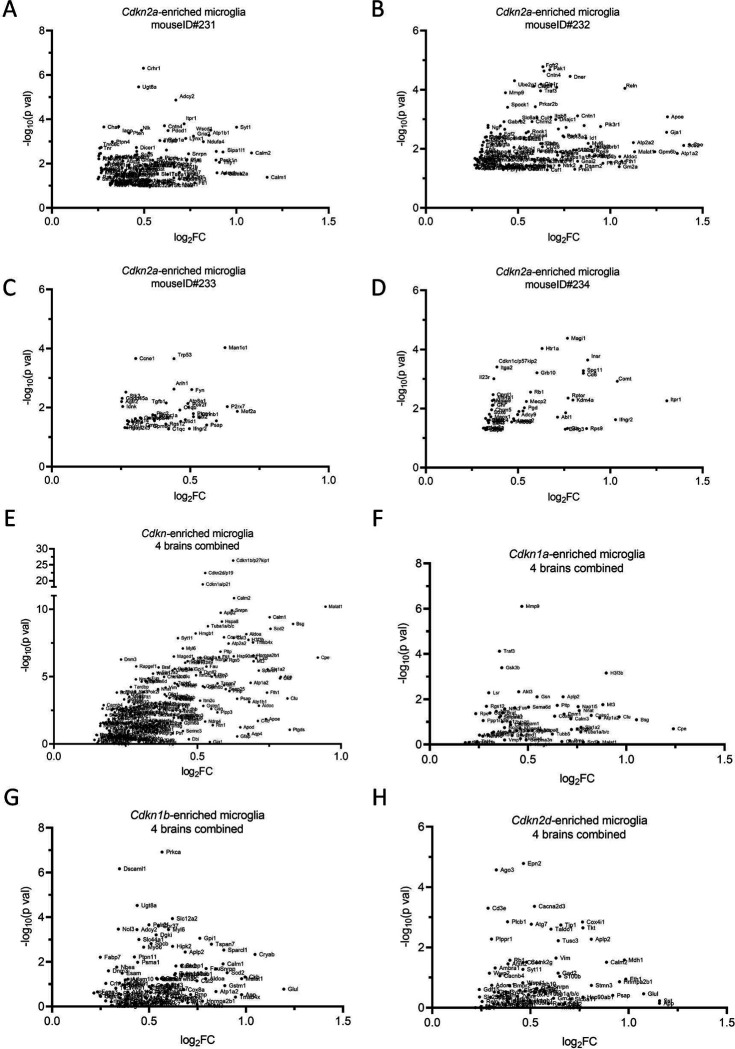
CosMx reveals conservation and heterogeneity of *Cdkn*+ single-cell gene expression profiles across aged brain samples. Related to figure 4 and 5: Transcript volcano plots derived from *Cdkn2a-enriched* microglia clusters are separated by each mouse brain (A)ID#231, (B)ID#232, (C)ID#233, (D)ID#234. (E) Transcript volcano plot from microglia pooled from four aged brains enriched for all cyclin-dependent kinase inhibitors, with the criteria of at least two or more transcripts detected within a cell. Transcript volcano plots from pooled microglia were parsed for cells enriched for (F) *Cdkn1a*, (G) *Cdkn1b*, and (H) *Cdkn2d*.

We also explored the expression profiles of *Cdkn2a*-enriched microglia among the four merged and individual aged brains (Fig. 4N–O, Extended Data Figure 4). The overall signature was characterized by downregulation of *Sall1*, increased expression of synapse-associated *Atp2b2* and *Grin2b,* and increased *Foxj1*, which is expressed along the ventricular surface of the brain^40^ (Fig. 4O). Across the individual brains, we observed increased expression of senescence biomarkers including *Trp53*, *Ccne1*, *Cdkn1c*, *Pak1*, *Pdcd1*, and *Malat1* from *Cdkn2a*-enriched microglia (Extended Data Figure 4A–D). Microglia expressing other cyclin-dependent kinase inhibitor genes, including *Cdkn1a, Cdkn1b,* and *Cdkn2d* had diverse profiles which revealed both conserved and emerging markers of senescence (Extended Data Figure 4E–H).

**Extended Data Figure 5.**
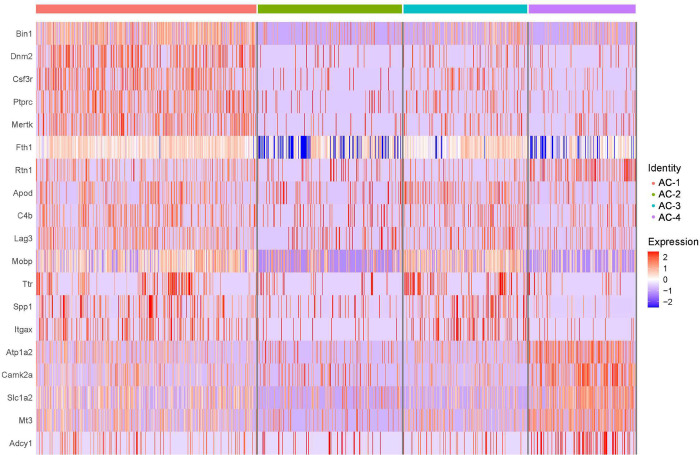
Top highly-expressed gene markers across pooled senescent and DAM-like microglia subclustered into AC-1, AC-2, AC-3, and AC-4. Related to figure 5. Single-cell heatmap of top five differentially genes expressed in each AC subcluster as detected by Wilcoxon rank sum test.

**Figure 5.**
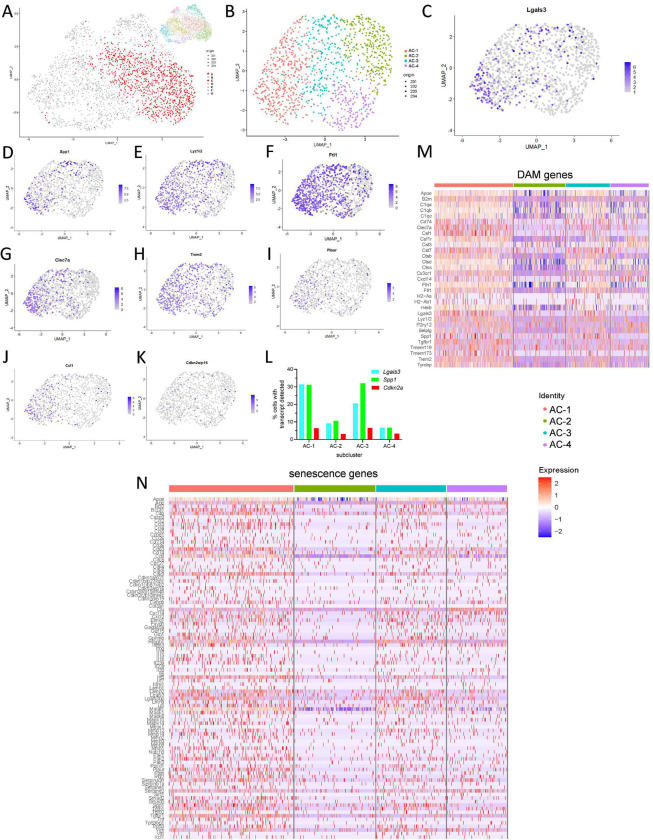
Microglia harbor both convergent and heterogenous DAM and senescent gene profiles across different old brains. (A) Pooled microglia UMAP as in Fig. 4H (also inset), selecting clusters A and C (red cells = cluster “AC”) for further subclustering based on high-density of DAM and senescence marker expression. (B) Cluster AC re-clustered by Leiden algorithm into subclusters AC-1, AC-2, AC-3, and AC-4 based on gene expression alone. (C-K) UMAP feature plots of Fig. 5B by normalized expression of single genes of interest (C) *Lgals3*, (D) *Spp1*, (E) *Lyz1/2*, (F) *Ftl1*, (G) *Clec7a*, (H) *Trem2*, (I) *Plaur*, (J) *Csf1*, and (K) *Cdkn2a*, showing highest concentration of DAM and senescence genes in subcluster AC1. (L) Percentage of cells with transcripts detected for each of the genes *Lgals3* (cyan), *Spp1* (green), and *Cdkn2a* (red) in each of the four subclusters AC-1, AC-2, AC-3, and AC-4, as in B. (M-N) Single-cell heatmaps of (M) DAM and (N) senescence genes of normalized and scaled data, organized by AC subclusters.

Based on enriched senescence and DAM profiles, we combined and re-clustered pooled clusters A and C, which generated four DAM-specific subclusters AC-1, AC-2, AC-3, and AC-4 (Fig. 5A–B). *Lgals3* was most concentrated in subcluster AC-1 (Fig. 5C), with an overlapping profile of *Spp1* (Fig. 5D)*, Lyz1/2* (Fig. 5E)*, Ftl1* (Fig. 5F)*, Clec7a* (Fig. 5G)*, Trem2* (Fig. 5H)*, Plaur* (Fig. 5I), and *Csf1* (Fig. 5J). *Cdkn2a*-expressing microglia were scattered throughout the DAM subclusters (Fig. 5K). Subcluster AC-1 microglia were characterized by overall greater expression of DAM and senescence genes (Fig. 5L–N; Extended Data Figure 5). Collectively, CosMx profiling enables high-dimensional mapping of DAM and senescent microglia, which are enriched in hippocampal-adjacent white matter. Merging of biological replicate data demonstrates the reproducibility, conservation, and heterogeneity of cell profiles.

### Age-dependent increases in *Lgals3* and *p16*^*ink4a*^ expression in white matter are suppressed by senotherapeutic interventions

**Figure 6.**
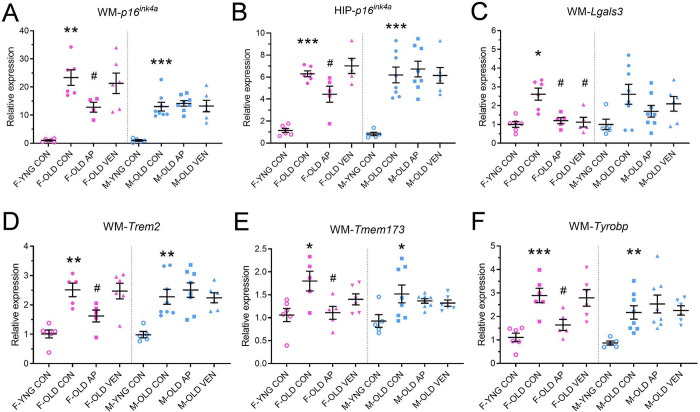
Senotherapeutics reduce senescence and DAM gene expression in aged white matter. Summarized are (A, C-F) white matter (WM) and (B) hippocampal (HIP) gene expression values from female (pink) and male (blue) mice in comparison of young vehicle-treated (YNG CON, open circles), old vehicle-treated (OLD CON, closed circles), old AP20187-treated (OLD AP, closed squares), and old venetoclax-treated (OLD VEN, closed triangles) groups. Values are normalized relative to the expression of sex-matched YNG CON. (A-B) *p16*^*ink4a*^, (C) *Lgals3*, (D) *Trem2*, (E) *Tmem173*, (F) *Tyrobp*, gene expressions were quantified (one-way ANOVA, * p < 0.05, ** p < 0.01, *** p < 0.001: OLD CON vs. YNG CON; # p < 0.05 vs. OLD CON, n = 4–8 mice). Bars represent mean ± S.E.M.

We next tested the hypothesis that systemic targeting of senescent cells would reduce the abundance of canonical senescence and DAM markers, including *p16*^*ink4a*^ and *Lgals3*, in aged white matter. We treated old *p16-InkAttac* mice with vehicle, AP20187, which induces cell death in p16-expressing cells^41^, or the senolytic BCL2-inhibitor venetoclax, based on our confirmation of *Bcl2* expression in senescent DAM (Fig. 4E–F)^20^. Young vehicle-treated mice were also assessed. AP20187 treatment significantly reduced *p16*^*ink4a*^ expression in female white matter (Fig. 6A) and hippocampus (Fig. 6B). AP20187 and venetoclax significantly reduced age-associated *Lgals3* in female white matter (Fig. 6C), and DAM genes *Trem2*, *Tmem173*, and *Tyrobp* were reduced by AP20187 in female white matter (Fig. 6D–F). Transcriptional profiles were comparatively more heterogenous in males, with DAM genes increasing in white matter in aged males. Based on our observations that senotherapeutic drugs reduced *p16*^*ink4a*^, *Lgals3,* and DAM gene expression, we next explored whether they modulated aged white matter microglial identity, frequency, morphology, and distribution.

### Senotherapeutics shift aged fimbria microglia identity and abundance to a more youthful state

**Figure 7.**
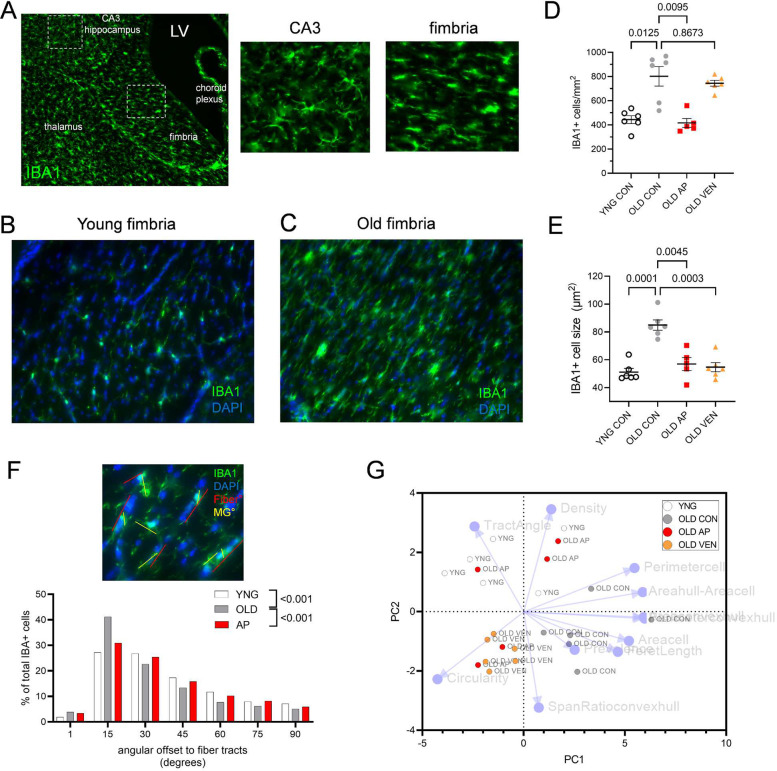
Senotherapeutics alter white matter fimbria microglia density, morphology, and organization. (A) Immunolabeling of IBA1 in fimbria of an old mouse sagittal brain section. Increased magnification of distal CA3 (middle panel) and fimbria (right panel); IBA1+ cells exhibit region-specific morphology. IBA1+ (green) and DAPI (blue) counterstains in fimbria demonstrate (B) young microglia with ramified processes and (C) old microglia exhibiting spindle-like or enlarged cell soma morphologies. (D) Summarized quantification of IBA1+ cell density (per mm^2^) in young vehicle-treated (YNG CON), old vehicle-treated (OLD CON), old AP20187 treated (OLD AP), and old venetoclax-treated (OLD VEN) fimbria. (E) Summarized quantification of IBA1+ cell size (μm^2^) in YNG CON, OLD CON, OLD AP, and OLD VEN fimbria (D-E, p values denote one-way ANOVA with multiple comparisons correction, n = 5–6 mice. Bars represent mean ± S.E.M). (F, top) Representative IBA1+ (green) and DAPI (blue) counterstain of fimbria and oligodendrocyte fiber tracts with computed angle of the fiber tract (red) and angle of longest feret axis of each IBA1+ cell. (F, bottom) Summarized histogram of measured angular offset of IBA1+ cells to oligodendrocyte fiber tracts from YNG, OLD, and AP20187-treated (AP) fimbria (p-value denotes Kolmogorov-Smirnov test vs. OLD, KS_D_(YNGvOLD) = 0.1686, KS_D_(APvOLD) = 0.1098). (G) Principal component analysis of morphological features of IBA1+ cells showing group-specific clustering of YNG, OLD CON, OLD AP, and OLD VEN fimbria. Each point represents one mouse.

We investigated the impact of senotherapeutic interventions on myeloid cell properties in the fimbria. Using the opposite hemisphere of brains applied to gene expression profiling above (Fig. 6), we conducted IBA1+ immunofluorescent imaging of microglia in young, old, old AP20187-treated, and old venetoclax-treated *p16-InkAttac* female mice (Fig. 7). Compared to ramified microglia found in the hippocampus, fimbria-resident IBA1+ cells exhibited more frequent spindle- or amoeboid-like morphologies (Fig. 7A, 7C). We observed greater density of IBA1+ cells in old fimbria as compared to young (Fig. 7B–D), and old IBA1+ cells had larger cell area (Fig. 7E). AP20187-treatment in old mice reduced the density of IBA1+ cells in the fimbria to youthful levels (Fig. 7D). AP20187 or venetoclax treatment decreased IBA1+ cell size, compared to old controls (Fig. 7E). We explored microglia organization to fiber tract architecture by analyzing the long-axis angular offset of each IBA1+ cell to the nearest oligodendrocyte tract. Microglia from old mice displayed greater alignment and proximity to fiber tracts than young controls, but AP20187 treatment shifted angular offset of microglia toward the more youthful organization of fimbria-resident microglia (Fig. 7F).

We applied an established microglial phenotyping composite index^42^ through principal component analysis (PCA) using properties of fimbria IBA1+ cells across the experimental conditions (Fig. 7G, **Extended Data Table 4**). Old control microglia were distinct from young, old AP20187, and old venetoclax microglia as defined by morphometric properties of larger cell size. Microglia from AP20187-treated mice shifted toward the principal component profiles of young mice, associated with cell density and tract-angle offset properties. Microglia from venetoclax-treated mice and two AP20187-treated mice clustered tightly as mainly defined by increased circularity of the cell and decreased cell size. Therefore, morphological properties of aged microglia in fimbria are modulated by senotherapeutic interventions.

### Senotherapeutic interventions suppress DAM abundance and organization in aged fimbria

**Extended Data Figure 6.**
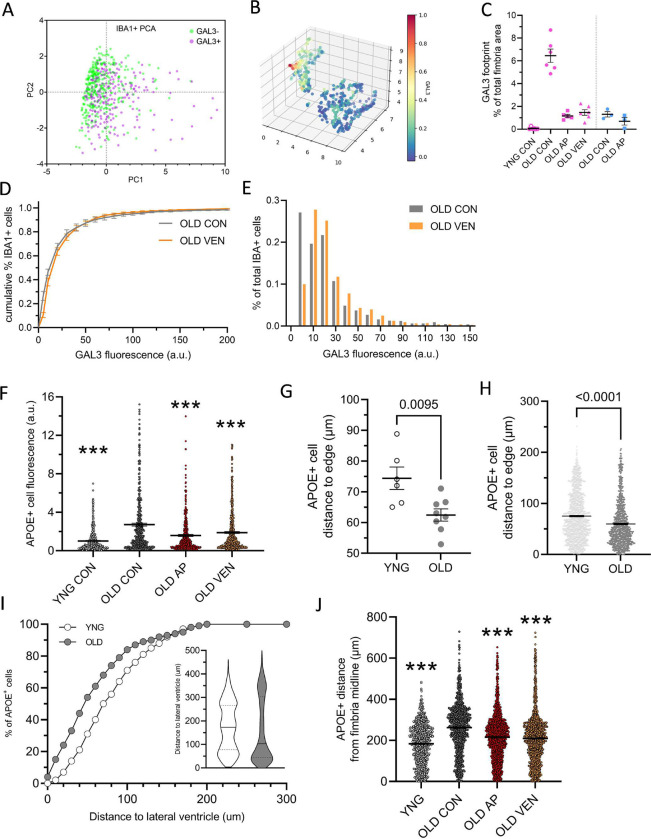
Senotherapeutics modulate fimbria microglial identity and organization. Related to figure 8: (A) Principal component analysis (PCA) of morphological features of aged IBA1+ cells that are classified by GAL3− (green circles) or GAL3+ (purple squares) immunoreactivity, as in Fig. 8B. Cells were categorized by GAL3 based on thresholding derived from young GAL3− microglia. (B) UMAP of GAL3-stratified immunofluorescence intensity of each cell, derived from PCA as shown in A. (C) GAL3 immunofluorescence signal as percentage of total fimbria area measured for female (pink) and male (blue) mice. (D) Cumulative distribution plot of IBA1+ cell populations according to each cell’s GAL3 immunofluorescence intensity from old vehicle-treated (OLD CON, grey) and old venetoclax-treated (OLD VEN, orange) groups. (E) Histogram depiction of the same GAL3 intensity data as in D. (F) Cell populations for each experimental group YNG CON, OLD CON, OLD AP, (OLD VEN) in quantification of APOE+ cell fluorescence, as in Figure 8F–G. n = 500 cells per group. *** p < 0.001 vs. OLD CON, one-way ANOVA with multiple comparisons. (G-H) Summarized mean distance of APOE+ cells to nearest edge of the fimbria (G) per mouse (n = 6–8 mice) and (H) per cell population (n = 863 – 1083 cells). p value denotes unpaired t-test of YNG vs. OLD. (I) Cumulative distribution plot of APOE+ cell populations distance to lateral ventricle edge. Inset shows violin plot of group data. (J) Summarized group populations of APOE+ cell distance from fimbria midline. *** p < 0.001 vs. OLD CON, one-way ANOVA with multiple comparisons. n = 1113 – 1759 cells. Bars represent mean ± S.E.M.

**Figure 8.**
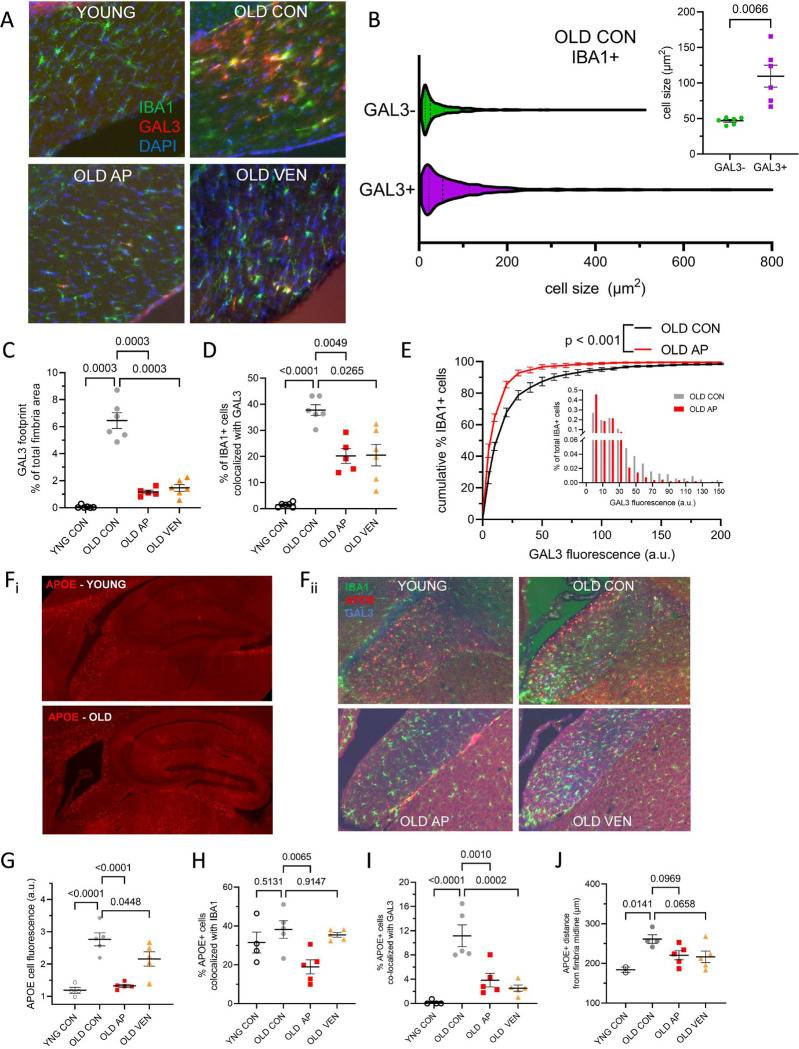
GAL3+ and APOE+ microglia in the aged fimbria are sensitive to senotherapeutic modulation. (A) Representative images of IBA1+ (green) and GAL3+ (red) cells co-stained with DAPI in the fimbria from young vehicle-treated (YNG CON), old vehicle-treated (OLD CON), old AP20187-treated (OLD AP), and old venetoclax-treated (OLD VEN) mice. (B) Violin plot of the cell size (μm^2^) of IBA1+GAL3− (n = 1034 cells) and IBA1+GAL3+ (n = 740 cells) cells from OLD CON female mouse fimbria. Dashed lines represent population quartiles. Inset shows direct quantification of mean cell size of GAL3− (green) and GAL3+ (purple) IBA1+ cells (paired t-test, mean ± SEM, n = 6 mice). (C) Summarized quantification of GAL3 immunoreactivity in fimbria as percentage of total fimbria area and (D) percentage of IBA1+ cells colocalized with GAL3, in female YNG CON (open circles), OLD CON (grey circles), OLD AP (red squares), and OLD VEN (orange triangles) groups (C-D, p values denote one-way ANOVA with multiple comparisons correction, n = 5–6 mice). (E) Cumulative distribution plot of IBA1+ cell populations according to each cell’s GAL3 immunofluorescence intensity from OLD CON (grey) and OLD AP (red) groups. Inset shows histogram depiction of the same data. p value denotes Kolmogorov-Smirnov test vs. OLD CON, KS_D_ = 18.2, n = 6 mice per group. (F_i_, left) Sagittal sections of hippocampus and adjacent white matter APOE immunofluorescent staining in representative young (top) and old (bottom) mice. (F_ii_, right) Representative immunofluorescence images for IBA1 (green), APOE (red), and GAL3 (blue) in experimental mice. (G) Summarized quantification of APOE+ cell fluorescence intensity per group. (H-I) Summarized quantification of percentage of APOE+ cells colocalized with (H) IBA1 or (I) GAL3. (J) Summarized quantification of distance between APOE+ cells and midline of fimbria in two spatial dimensions. p-values denote one-way ANOVA with multiple comparisons correction. n = 4–5 mice. Bars represent mean ± S.E.M.

We hypothesized that GAL3+ microglia are targeted by senotherapeutics. The rationale was based on the observations that 1) *Lgals3* transcript and GAL3 protein accumulate in aged white matter, 2) *Lgals3-*expressing microglia are enriched with a senescence gene profile, and 3) senotherapeutic interventions reduced DAM and senescence gene expression in white matter. Accordingly, we tested whether senotherapeutic drugs alter IBA1+GAL3+ cell morphology and frequency (Fig. 8A). In old female brains, IBA1+GAL3+ cells were larger size relative to IBA1+GAL3− cells (Fig. 8B). IBA1+GAL3− cells from old mice were similar in size to IBA1 cells from young mice, suggesting that the age-dependent increase in IBA1+ cells described previously (Fig. 7) corresponds to IBA1+GAL3+ microglia. PCA of IBA1+GAL3+ and IBA1+GAL3− populations revealed size-specific principal components on the basis of cellular morphology and GAL3+ immunoreactivity (Extended Data Figure 6A–B). In old fimbria from females treated with vehicle, GAL3+ cells occupied greater area compared to young females (Fig. 8C). AP20187 or venetoclax treatment reduced the frequency of IBA1+GAL3+ cells in aged female fimbria (Fig. 8C–D). In old male fimbria, we identified significantly lower GAL3+ immunoreactivity as compared with old females (Extended Data Figure 6C). IBA1+ cells from AP20187-treated females also had decreased GAL3+ immunofluorescence intensity compared to old controls (Fig. 8E). Whereas venetoclax decreased *Lgals3* transcript and reduced the total number of IBA1+GAL3+ cells in white matter (Fig. 8D), we did not detect differences in GAL3+ immunofluorescence intensity of IBA1+ cells in fimbria in venetoclax-treated aged females (Extended Data Figure 6D–E), suggesting a different effector population of venetoclax treatment as compared to *p16-InkAttac* targeting with AP20187.

Based on our aged fimbria GeoMx and CosMx profiles demonstrating pronounced colocalization of *Apoe* expression in *Lgals3+* or GAL3+ microglia, we performed immunostaining for APOE and GAL3 (Fig. 8F). We observed high density of APOE+ cells in fimbria and corpus callosum as well as colocalization of APOE and GAL3, but APOE+ cells were seldom found in the hippocampus. Old control mice had significantly greater immunofluorescence intensity than young, and AP20187 and venetoclax both reduced overall APOE signal (Fig. 8F–G, Extended Data Figure 6F). AP20187 treatment significantly reduced the percentage of APOE cells colocalized with IBA1 (Fig. 8H). Both senolytic treatments significantly reduced the percentage of APOE+ cells colocalized with GAL3 (Fig. 8I). Interestingly, we observed a distinct APOE+ architectural pattern in old versus young white matter, and we quantified the frequency of APOE cells relative to the midline of the fimbria in two dimensions. We discovered that old animals had pronounced lateral localization of APOE cells to the lateral ventricle compared to young. AP20187- or venetoclax-treated mice exhibited a more dispersed population characterized by trends toward decreased distance to the midline, relative to old vehicle-treated mice (Fig. 8F–J, Extended Data Figure 6G–J). Collectively, treatment with the senotherapeutic interventions reduced DAM and reverted microglial properties in the aged fimbria to more youthful states.

## DISCUSSION

White matter integrity is a key correlate of neuronal activity and cognitive function^43, 44^. In mice and humans, white matter tracts are selectively vulnerable in aging^5,31,45,46^. Although the underlying cellular and molecular factors are not well understood, inflammatory activation and maladaptive microglial changes are emerging as stereotypic features underlying white matter aging^30,31,47^. We applied gold-standard imaging and gene expression analyses together with innovative spatial molecular profiling to map cell-type specific morphological, molecular, and functional features of microglia in aged hippocampal gray matter and adjacent white matter. Our results reveal previously unachieved molecular and spatial diversity in limbic microglial states and implicate GAL3/*Lgals3* as a key senescence- and DAM-linked biomarker of aged white matter. We demonstrate that systemic senolytic targeting of *p16* or BCL2 has profound effects on microglia identity and organization in aged brain white matter.

Microenvironment and diverse functional demands throughout the lifespan contribute to heterogeneity across brain cell types, imposing challenges in the identification of cell fates that mechanistically contribute to dysfunction, including DAM and senescent cells. We previously showed that senescence- and inflammation-related expression profiles differed across aged brain regions and identified an overlapping cell signature of senescence and DAM that accumulates in the aged brain^20^. Herein, we discovered that isolated hippocampus (devoid of large white matter fiber bundles) harbored lower expression of senescence and DAM genes, relative to adjacent white matter, which we confirmed by immunofluorescent imaging.

To build on these findings, we conducted integrated spatial mapping of aged brain. Testing of three platforms, IMC, GeoMx digital spatial profiling, and CosMx spatial molecular imaging permitted exploration of distinct and conserved features of microglia in hippocampal gray versus white matter, while revealing inherent strengths and limitations of each technology. IMC enabled us to validate the immunofluorescent imaging observation that GAL3+ cells are more abundant in fimbria, relative to hippocampus. We also showed that fimbria harbored higher levels of senescence-related proteins, including UPAR, dPP4, and CD38, and these proteins were enriched in GAL3+ microglia. A strength of IMC is the ability to profile up to 40 proteins simultaneously with minimal signal overlap; however, challenges include reliance on customized antibody panels. Through IMC, we offer new functional indicators of senescence-related GAL3+ microglia in aged fimbria.

We employed a whole-transcriptome mapping strategy, GeoMx digital spatial profiling, to interrogate IBA1+ microglial gene signatures in the fimbria and hippocampus. We discovered both conserved DAM and novel genes were enriched in the fimbria. However, *Apoe* uniquely distinguished GAL3+ versus GAL3− microglia in the fimbria, a comparison comprised of fewer total cells relative to the area comparisons. These experiments emphasize the utility of this method for discovery-based mapping of transcriptional profiles and underscore the requirement of profiling sufficiently large cellular areas for discovery of novel differentially expression patterns. Nevertheless, our use of GeoMx substantiated enrichment of DAM signatures in fimbria, including the canonical DAM factor *Apoe*, and revealed new genes of interest in aged fimbria versus hippocampus.

Through CosMx spatial molecular imaging, we mapped microglial spatial-transcriptional profiles at single-cell resolution across the hippocampus and adjacent white matter. This powerful method enabled several conclusions. First, distinct gene profiles demarcated aged white versus gray matter-localized microglia. Second, senescence and DAM molecular signatures were identifiable across aged microglia subpopulations but were most pronounced in a white matter-localized subpopulation that was rarely identified in gray matter. Third, through population and single-cell analyses, we discovered that a dominant senescent DAM population expressed a pattern of canonical and novel markers, including *Apoe, Bcl2, Ccl3, Ccl4, Cdk9, Cdkn1b, Cdkn2b, Cdkn2d, Gdf15, Gpnmb, Lgals3, Pdcd1, Plaur, Sirt1, Spp1, Tgfb*, and *Trp53*. Finally, based on regional expression results, we hypothesized that senescent DAM microglia coexpress key biomarkers, *Cdkn2a/p16*^*ink4a*^ and *Lgals3*. Surprisingly, CosMx profiling revealed expression of these markers in aged microglia characterized by close proximity and/or overlapping gene profiles but with rare coexpression, collectively indicating that *Cdkn2a-* and *Lgals3-*defined cells are related and may interact but are distinct. By comparing *Cdkn2a-* and *Lgals3*-specific gene signatures across four aged brains, we discovered a highly-conserved *Lgals3* gene profile and a more heterogenous *Cdkn2a* gene profile. The increased heterogeneity may correspond to lower overall frequency of *Cdkn2a*+ cells, relative to *Lgals3*+ cells, but it also highlights that senescence profiles in aging are inherently variable. We also demonstrate dynamic expression of other putative senescence markers, including *Cdkn1b, Cdkn2b,* and *Cdkn2d*; the functional roles of these factors in aged microglia warrant further investigation. Multi-omic integration of spatial datasets is another important future direction. We discovered that senescence gene *Plaur*, which encodes UPAR^48^, increased in CosMx DAM profiles coexpressing *Lgals3* and other senescence and DAM genes, and UPAR increased in IMC-identified GAL3+ aged fimbria microglia, ultimately confirming a white-matter enriched senescent biomarker revealed through comparative IMC and CosMx profiling. The signatures characterized here establish feasibility for mapping senescent identities across brain cell types and regional microenvironments in aging and disease states.

Our findings build on growing literature demonstrating changes in white matter as early and pronounced features of brain aging. We speculate the fimbria’s susceptibility to inflammatory changes in aging arises from biochemical and structural factors. Aside from the fimbria’s physical connection to the hippocampus as a primary tract of efferent and afferent projections, the fimbria is spatially adjoined by choroid plexus and the lateral ventricle and therefore, may experience greater exposure to the influence of systemic circulating factors and infiltrating immune cells^49,50,51^. Based on our novel findings herein, we posit that the anatomical location of the fimbria may underlie both its unique vulnerability in aging and its responsiveness to senotherapeutic intervention. Structural and pharmacokinetic parameters of the drugs tested here suggest that venetoclax may more directly affect the brain, relative to AP20187, and yet, we discovered that both senescence-targeting agents influenced aged fimbria molecular and cell fates through distinct and overlapping profiles. Whether and how these drugs variably penetrate aged brain regions to directly act on parenchymal cells versus how the drugs may modulate circulating, ventricular, and/or choroid plexus cells and molecules to indirectly influence parenchymal cells are important open questions.

Collectively, this research illuminates fundamental aging cell fates that contribute to white matter remodeling. Through use of established and emerging imaging and molecular methods, we demonstrate pronounced senescence and DAM cellular signatures in aged white matter, which coordinate tissue remodeling and may underlie neuropathological cascades eventuating in cognitive decline^32,43,52^. Our results demonstrate that systemic BCL2 or *p16*-targeting are effective strategies to counter DAM and senescence profiles in aged white matter and revert molecular and cellular organization to a more youthful state.

## METHODS

### Animals

Mouse experiments were performed under protocols approved by Mayo Clinic Institutional Animal Care and Use Committee. Male and female heterozygous *p16-InkAttac* mice (C57BL/6 background) were used in this study, and sex-specific features of age were analyzed independently. Mice were group-housed in ventilated cages with a constant temperature of 25°C, 30–70% humidity, a 12-hour light/dark cycle, and provided standard chow diet and water *ad libitum*. At the time of tissue collection for expression and treatment studies, young mice were 3–4 months of age and old mice were 16–20 months old. Alternatively, C57BL/6 were acquired from the National Institute of Aging and brains collected at 24–28 months of age for spatial profiling experiments. Mice were euthanized with a lethal dose of pentobarbital. Mice were transcardially perfused with ice-cold PBS. All mice were examined for gross pathology and tumor prevalence, with exclusion of mice harboring pronounced splenomegaly. Left hemisphere brain cortex, cerebellum, hippocampus, and hippocampus-adjacent white matter were immediately removed and placed in TRIzol for gene expression analyses. Right hemisphere brain was drop-fixed in 4% PFA for 24 hours. Brains used for traditional immunofluorescent imaging were transferred to 10% w/v sucrose in PBS for 24 hours, then 20% sucrose for 24 hours, then 30% sucrose for 48 hours, then cryopreserved in 30% glycerol, 30% ethylene glycol, 40% PBS w/v at −80°C. Brains used for imaging mass cytometry and spatial-omics profiling were removed from 4% PFA to cold 70% ethanol and embedded within paraffin wax within 24 hours for PPFE processing, cut at 5 μm slices with sliding microtome.

### Senotherapeutic interventions

For AP20187 drug dilution, a 12.5 mg/mL stock solution in 100% ethanol was created and stored at −20°C. One mL AP20187 working solution (2 mg/kg) was prepared by mixing 40 μL stock solution with 100 μL PEG-400 and 860 μL of 2% Tween-20 in ddH_2_O. The solution was vortexed and administered to mice by intraperitoneal (i.p.) injection within 30 minutes of diluting the stock. The corresponding vehicle was composed of ethanol, PEG-400, and Tween-20 and was administered to control animals via i.p. injection. For venetoclax drug dilution, a stock oral gavage vehicle was created as a solution of 60% phosphatidylcholine in propylene glycol (Phosal 50 PG, Lipoid GmbH), 30% PEG-400, and 10% pure ethanol. Venetoclax was freshly vortexed into vehicle solution at room temperature to reach a working solution of 50 mg/kg and stored at 4°C the week of injections. Old *p16-InkAttac* mice were randomized (based on body weight and age, 16–20 months old) to receive vehicle, AP20187 (2 mg/kg, i.p. injection), or venetoclax (50 mg/kg) by oral gavage. All mice received both gavage and i.p. treatments, with drug treatment groups also receiving vehicle by i.p. or by gavage. Young and old *p16-InkAttac* control mice received the same vehicle dosing strategy. Venetoclax (ABT-199, #A8194) was acquired from APExBio Technology. AP20187 was acquired from WuXi AppTec. All other reagents, unless otherwise denoted, were acquired from Sigma-Aldrich/Millipore. Mice received five consecutive daily administrations for one week, two weeks off, and then another five-day treatment. Treatments were discontinued one week prior to necropsy. Groups consisted of 5–8 mice per sex and treatment.

### Real-time polymerase chain reaction (RT-PCR)

Tissue samples collected at necropsy were immediately stored in TRIzol reagent. RNA was isolated using TRIzol-based chloroform-isopropanol precipitation, followed by nanodrop concentration and purity analysis. For all tissues, 2 μg of total RNA was used for cDNA synthesis through M-MLV reverse transcription (Invitrogen, cat# 18091200). RT-PCR was performed on a QuantStudio5 RT-PCR system (ThermoFisher) with PerfeCTa FastMix II Low ROX (Quantabio) and Taqman PrimeTime qPCR assays from Integrated DNA Technologies (IDT) (**Extended Data Table 1**). Genes were normalized to *Hprt* housekeeper gene expression. Relative gene expression compared to the young control group was derived from the 2^−ΔΔCT^ value for each tissue sample.

### Immunofluorescence imaging

Right hemisphere brains were embedded in Tissue-Tek O.C.T. compound and sagittally sliced at 30 μm section thickness on a Leica CM3050 S cryostat (Leica Biosystems). Free-floating sections were stored in wells containing ice-cold PBS + 0.01% sodium azide. Sections were blocked and permeabilized in 8% donkey serum, 0.1% Triton-X, 0.1% Tween-20 for 2 hours at 25°C. After 5× 5 min. thorough washes in cold PBS, sections were quenched for 10 min. with 1X True Black Plus lipofuscin autofluorescence quencher (Biotium, catalogue #23014), diluted from a 40X stock in PBS. After 5× 5 min. thorough washes in cold PBS, sections were stained with primary antibodies diluted in 8% donkey serum, overnight at 4°C. Antibody combinations consisted of: rabbit polyclonal anti-IBA1 (Fujifilm Wako, #019-19741, RRID:AB_839504) or goat polyclonal anti-IBA1 (Abcam, #ab5076, RRID:AB_2224402), rat IgG_2A_ monoclonal anti-galectin-3 clone eBioM3/38 (Invitrogen, #14-5301-85, RRID:AB_837133), goat polyclonal anti-GFAP (Abcam, #ab53554, RRID:AB_880202), or rabbit polyclonal anti-apolipoprotein E clone EPR19392 (Abcam, #ab183597, RRID:AB_2832971). The next day, after 3× 5 min. washes with PBS, sections were stained with donkey host secondary antibodies conjugated to fluorophores for 2 hours at 25°C (Jackson Immunoresearch, **Extended Data Table 5**). After 3× 5 min. washes with PBS to remove excess fluorophore, sections were mounted onto Superfrost Plus microscope slides with Vectashield with DAPI (4′,6-diamidino-2-phenylindole, Vector Laboratories, #H-1200) with a 1.5 glass coverslip. Sections were imaged with a Nikon Ti2 Eclipse Inverted microscope with 10X, 20X, and 40X Plan Apo objectives, 8-channel Spectra III light engine, and using the Orca Fusion BT sCMOS camera with Nikon Elements AR software. 405, 488, 594, and 647 laser lines were used with an image exposure time of 200 ms per channel in 16-bit readout mode. For each mouse, 3–6 sections were analyzed for each immunolabel, and 3–5 mouse brains were collected for each sex and group. ImageJ was used to process fluorescence intensity and colocalization image data. Total corrected cellular fluorescence intensity was calculated from the mean integrated density − (mean local background × cell area). Fluorescence was normalized to controls, scaled per channel, and is represented by arbitrary units. Alignment of microglia to oligodendrocyte fiber tracts was performed by calculating the feret length, the longest axis of each IBA1+ cell, and calculating the most acute angle to the direct X-Y line of the nearest fiber tract of linear nuclei within the same plane, to a maximum of 90°. The DiAna ImageJ plugin tool was used for spatial analyses of cell-cell objects and cell-edge proximities^53^.

### Imaging mass cytometry

The right hemispheres of aged female mouse brains (23–24 months) were harvested and placed in a 4% paraformaldehyde solution for 24 hours, then transferred to 70% ethanol. Formalin-fixed, paraffin-embedded sagittal sections (5 μm) were prepared by the Mayo Clinic Pathology Research Core. FFPE slides were baked at 60°C for 45 minutes. Slides were deparaffinized by submerging in xylene (3 times for 5 min.), 100% ethanol (2 times for 1 min.), 95% ethanol (2 times for 1 min. each), and 70% ethanol (1 time for 1 min.). For antigen retrieval, the slides were incubated in 1X Epitope Retrieval Solution (Leica Biosystems) at 97°C for 20 min. This was followed by a 5 min. wash with ultrapure water for rehydration. Sections were outlined with a hydrophobic barrier on the slide. The tissues were then incubated in Superblock Blocking Buffer (Thermo Fisher) at 25°C for 30 min, followed by a wash step with PBS-TB (3× 5 min.). The mass-tagged antibody cocktail (**Extended Data Table 5**) was applied to the sections and incubated overnight at 4°C in a hydration chamber with slight agitation. Immunofluorescent imaging of free-floating sections was performed to validate the binding of antibodies included in the panel for imaging mass cytometry. After overnight staining, slides were washed with PBS-TB (3 times for 5 min. each) and stained with an iridium nucleic acid intercalator solution (1:400 in PBS-TB) at 25°C for 30 min. A wash step with PBS-TB (5 min.) was performed, followed by air-drying at 25°C for 20 min. The Helios time-of-flight mass cytometer (CyTOF) was used to laser-ablate the tissue within pre-selected regions of interest at a 1 μm resolution, followed by a plasma-based ionization step. The resulting signals from each metal-conjugated antibody, distinguished by CyTOF, were acquired by the Hyperion Imaging System (Standard BioTools), reconstructed into an image. Representative images were converted to OME.TIFF files using MCD Viewer software (Standard BioTools). Single microglia cells were isolated and segmented on CX3CR1 and IBA1 combined channels. Values were collected for each channel for each segmentation selection. Average local background was subtracted from the average intensity to derive cellular protein levels for each antibody label.

### GeoMx digital spatial profiling

Five μm sagittal sections were sliced from 28-month female mouse brain PPFE blocks and mounted on Superfrost Plus slides. Slides were baked at 60°C for 30 minutes, followed by deparaffinization with xylene (3 × 5 min. washes), 100% EtOH (2 × 5 min. washes), 95% EtOH (1 × 5 min. wash), and PBS (1 × 1 min. wash). Slides were steamed for 15 min. at 99°C in 1X Tris EDTA Epitope Retrieval Solution, pH 9 (Leica,# RE7119-CE) followed by a 5 min. wash in PBS. Slides were incubated in 1 μg/mL proteinase K for 15 min. at 37°C and washed in PBS for 5 min. 200 μL mouse whole-transcriptome hybridization solution containing GeoMx RNA probe mix (Nanostring) was applied to each slide and incubated 37°C overnight in a hybridization chamber. Slides were then incubated in Stringent Wash Solution at 37°C for 2 × 25 min. washes, followed by 2 × 2 min. washes in SSC-T solution. Slides were blocked with GeoMx Buffer W for 30 min., quenched with True Black as above, and immunostained with the morphology markers anti-IBA1, anti-GAL3, and anti-GFAP, and SYTO83 as nuclear stain. Secondary antibodies were subsequently added as anti-rabbit AF488, anti-rat AF594, and anti-goat AF640. Slides were washed 2× 5 min. with SSC prior to processing in GeoMx profiler. n = 5 mice, 2–3 sections per mouse.

Areas of interest (AOI) were selected from cells in the hippocampus and fimbria for IBA1+SYTO83+, GAL3+SYTO83+ alone, and IBA1+GAL3+SYTO83+ cells, with GFAP as a negative-selection marker for each AOI. Segmentation order was 1) IBA+/GFAP−/GAL3−, 2) GAL3+/IBA1−/GFAPØ, 3) IBA1+/GAL3+/GFAPØ, with (+) indicating include, (−) indicating exclude, and (Ø) indicating ignore. Bound oligonucleotides were collected in a 96-well plate with annotation for mouse and section identities, brain structure of origin (hippocampus or fimbria), and AOI group. Primer libraries were created for mouse whole-transcriptome according to Nanostring GeoMx guidelines, and sequencing was performed on the Illumina System 2×150bp, ~350M PE reads (Azenta Life Technologies). The GeoMx NGS Pipeline was utilized to process FASTQ files and transform them into the Digital Count Conversion (DCC) file format, which can be read by the DSP instrument for subsequent analysis. The pipeline followed several key steps: (1) Selection of raw FASTQ files for a specific pipeline run. (2) Quality assessment of the selected reads. (3) Removal (trimming) of adapters and merging of paired-end reads to obtain high-quality reads. (4) Alignment of reads to the Readout Tag Sequence-ID (RTS-ID) barcodes. (5) Removal of PCR duplicates by matching based on the Unique Molecular Index (UMI). (6) Generation of the DCC file for further analysis. Genes of interest for differential comparison were selected with *a priori* threshold of 2 standard deviations greater than the geometric mean of the negative probe counts per AOI. A gene was filtered out if the percentage of AOIs surpassing the negative probe threshold was less than 15%. The combined gene list from each labeling condition group was then processed using 3^rd^ quartile normalization for differential expression. For hippocampus (n = 8 AOIs) versus fimbria (n = 15 AOIs) comparison of IBA1+ segments, each section was individually counted. For fimbria region comparison of IBA1+GAL3+ and IBA1+GAL3− segments, AOIs were pooled per mouse to achieve enough area from the sequencing. Differential expression analyses were conducted using the R package DESeq2 (version 1.36.0)^54^. As an exploratory method, genes were considered differentially expressed with a p-value < 0.05.

### CosMx spatial molecular imaging

CosMx spatial molecular imaging (Nanostring) was performed by RNA in situ probe hybridization as previously described^55^. We developed a 50-gene custom panel of senescence-associated genes, identified from SenMayo, SenSig, and other published gene panels, which we applied in tandem with the 950-plex mouse neuroscience panel for CosMx transcriptional mapping (**Extended Data Table 3**). In situ hybridization and four-marker antibody labeling (histone, rRNA, DAPI, GFAP) were performed on 5 μm PPFE sagittal sections from 24 month female mouse brains. Slides were baked at 60C for 30 min, and the CosMx Mouse Neuroscience Panel for RNA reagent application protocol was followed as specified in the user manual. 500 μm^2^ FOVs were collected in a gridded manner across a section of the brain including cortex, hippocampus, thalamus, and surrounding white matter. In situ transcript counts were derived from cell segments rendered from the antibody marker labeling. Nanostring AtoMx and the R packages Seurat (4.3.0)^56^ and Giotto Suite (3.3.0)^57^ were used for CosMx data analyses (see **Supplementary Information**). Cells with fewer than 20 total transcripts were excluded from further analyses. Transcript counts per gene were normalized to the total transcripts per cell and scaled. To visualize and delineate subpopulations of cells, highly variable features were derived from high coefficient of Pearson variance and PCA was generated. UMAP plots were then created from the PCA, followed by Leiden clustering across 1000 iterations. For a single brain processed through CosMx, microglia were selected as a dataset based on previously validated markers *Csf1r*, *Ctss*, *Cx3cr1*, *Hexb*, *Selplg*, *Itgam*, *P2ry12, Tmem119*, and *Trem2* ^38,39^. Macrophages were delineated out of the microglia cluster by detection of a specific gene matrix including concerted upregulation of genes *Crip1, Cd74, Fxyd5, H2-Aa, H2-Ab1, H3f3b*, and low expression of *Cd9* and *Ctsd* (**Supplementary Information**). All microglia were then processed for clustering via Leiden algorithm with resolution set to 0.1 and run through 1000 iterations. The Gini and Scran methods were used to detect differently expressed gene markers among the subclusters^58,59^. Differentially expressed gene markers were detected for each cluster relative to other microglia clusters. Single-brain normalized expression and cluster metadata were then converted into a Seurat object for integration into a merged object including four mouse brains. Scaling, detection of variable features, PCA, nearest-neighbor detection, and subclustering with Leiden algorithm were then processed for individual and pooled microglia single-cell datasets. Upon identification of *Lgals3*-enriched or *Cdkn2a*-enriched concentration of cells within the pooled dataset, the clusters were subset into a DAM-specific cluster for final detection of differentially expressed genes. Significantly expressed genes were represented as log_2_-transformed fold-change at an adjusted p-value < 0.05. Experiments were run with one technical replicate section each from four mice, #231, #232, #233, and #234, and a second biological replicate section from #231.

### Statistics

For RT-PCR data of old mouse brain hemisphere comparisons from Figure 1, each region was normalized to the relative expression of hippocampus alone. Among the three regions, they were compared for each sex by one-way ANOVA with Tukey’s test for multiple comparisons and significance denoted at p < 0.05. In comparisons of young and old RT-PCR data in Figure 1, values were normalized as relative expression to young and compared by unpaired t-tests with FDR multiple comparisons correction, *q < 0.05 vs. young tissue. For GeoMx data, differentially expressed genes were detected on FDR adjusted p-value < 0.05. For CosMx data, individual brain datasets were normalized and profiled by detection of differentially expressed genes by Gini and Scran methodologies. For pooled brain datasets in the Seurat pipeline, differentially expressed genes were detected by the Wilcoxon Rank sum test with significance set at an adjusted p value < 0.05. For RT-PCR, immunohistochemistry, and morphometric analyses across experimental groups in Figures 6–8, one-way ANOVA tests were performed with Dunnet’s test for multiple comparisons. Drug-treated groups were determined to be significantly different than the old vehicle-treated control group at adjusted p value < 0.05 with sample sizes of 5–8 mice per group. The cell fluorescence intensities for cumulative distribution populations of IBA1+ cells were compared by Kolmogorov-Smirnov test at p < 0.05. GraphPad Prism 9 and R 4.3.0 were used for statistical analyses.

## Extended Data

## Data Availability

The original contributions presented in the study are included in the article/supplementary information. Additional data included in this article are deposited in the NIH’s Cellular Senescence Network Consortium Organization and Data Coordinating Center (CODCC) and access to source data are provided with the Supplementary Information of this paper.
